# Ischemic Postconditioning Mitigates Retinopathy in Tree Shrews with Diabetic Cerebral Ischemia

**DOI:** 10.1155/2020/6286571

**Published:** 2020-02-12

**Authors:** Ling Zhao, Qiwei Liao, Yueting Zhang, Shufen Tan, Shuqing Li, Tingyu Ke

**Affiliations:** ^1^Department of Endocrinology, The Second Affiliated Hospital of Kunming Medical University, Yunnan 650101, China; ^2^Department of Cardiology, The Yan-an Affiliated Hospital of Kunming Medical University, Yunnan 650051, China; ^3^Department of Gynecologic Oncology, The Third Affiliated Hospital of Kunming Medical University, Yunnan 650101, China; ^4^Department of Pathophysiology, Kunming Medical University, Yunnan 650050, China

## Abstract

Ischemic postconditioning (PC) is proved to efficiently protect diabetic patients with acute myocardial infarction from ischemia-reperfusion injury. We aimed to explore the protective roles of ischemic PC on diabetic retinopathy in tree shrews with diabetic cerebral ischemia. A diabetic tree shrew model was established through high-fat diet feeding combined with streptozotocin (STZ) injection, while cortical thrombotic cerebral ischemia was induced photochemically. Tree shrews were divided into the normal control group, sham operation group, diabetes mellitus group, diabetes mellitus+cerebral ischemia group, and diabetes mellitus+cerebral ischemia+PC group (in which the tree shrews with diabetic cerebral ischemia were treated with ischemic PC). H&E staining was used to examine the pathological changes in the retina, and immunohistochemistry was performed to determine the retinal expression of VEGF (vascular endothelial growth factor). The modeling resulted in 77% tree shrews with diabetes. Ischemic PC reduced the blood glucose levels in the tree shrews with diabetic cerebral ischemia. Tree shrews with diabetes had thinned retina with disordered structures, and these pathological changes were aggravated after cerebral ischemia. The retinopathy was alleviated after ischemic PC. Retina expression of VEGF was mainly distributed in the ganglion cell layer in tree shrews. Diabetes and cerebral ischemia increased retinal VEGF expression in a step-wise manner, while additional ischemic PC reduced retinal VEGF expression. Therefore, ischemic PC effectively alleviates retinopathy in tree shrews with diabetic cerebral ischemia, and this effect is associated with reduced retinal VEGF expression.

## 1. Introduction

Diabetic retinopathy (DR) is one of the serious microvascular complications of diabetes mellitus (DM), and it is also a disease with a high incidence of blindness [1–3]. Approximately one-third of the people with diabetes have signs of DR, and one-third of DR cases develop a vision-threatening form of the disease. Diabetic patients have a disease course of more than 20 years, and more than 60% of them will develop retinopathy [4, 5]. Therefore, with the increasing incidence of DM, the incidence of DR and its blindness rate also show an increasing trend [6, 7]. Studies have shown that the blindness rate in patients with DR is 20 times higher than that in nondiabetic patients. In 2015, about 36 million of DR patients in the world became blind, and 216 million were suffering from impaired vision [3, 5]. Particularly, the population in the Asia-Pacific region is more common for diabetes-caused vision loss. Since DR is the leading cause of vision loss in adults [8], increasing attention has been drawn for the basic and clinical research on DR recently.

The pathogenesis of DR is very complex and multifactorial [9, 10]. Current studies have revealed that the incidence of DR is closely related to cytokine expressions in the retina. Among them, vascular endothelial growth factor (VEGF) plays an important role in the progression of DR [11–13]. VEGF is one of the main factors that promote neovascularization in the body, and only a small amount is expressed in the retinal ganglion cell layer of the normal body. However, under hyperglycemia conditions, retinal pericytes and vascular endothelial cells can express VEGF in a large amount due to retinal ischemia and hypoxia, which impairs the blood-retina barrier and results in increased vascular permeability and retinal edema [11, 12]. Excessive expression of VEGF can also lead to selective loss and degeneration of pericytes, which weakens the inhibition of endothelial cell proliferation and thus results in aggravation of the pathological process of DR due to capillary occlusion [11, 12]. Therefore, the retinal expression of VEGF is an effective parameter for evaluating the therapeutic effects of drugs and DR disease progression in animal models.

With an extremely complexed structure, the retina contains a large number of highly differentiated neurons. Since the metabolism in the retina is profoundly active, the tolerance of retinal cells to ischemia and hypoxia is poor. Therefore, the mechanisms of retinal ischemia and anti-injury protection have always been the hot topics in the field of DR pathogenesis [10, 14, 15]. Despite a large number of basic experiments and clinical studies at home and abroad, there is still no effective means of prevention, early intervention, and treatment of DR. However, the discovery of ischemic postconditioning (PC) has brought new hope to patients with ischemic cardiocerebral vascular diseases. Ischemic PC is a transient physiological phenomenon that helps to restore the blood flow through repeated opening and reclosing of the body tissues and organs immediately after ischemia. It is an endogenous anti-ischemia and anti-hypoxia protection mechanism that exists in different tissues and organs [16–18]. Since PC is an internal protective mechanism activated by ischemia, it is safer and more effective than exogenous drugs and exogenous ischemic preconditioning.

Multiple studies using rodent models have confirmed the protective roles of PC in DM progression [19–23]. More recently, limb remote ischemic conditioning has been demonstrated to significantly downregulate VEGF protein expression in a rat retina through the antioxidative and anti-inflammatory activities [24]. However, the similar protective roles of PC are not studies in models with primates so far. In this study, we used tree shrews (*Tupaia glis*), which have similar biological characteristics with monkeys, to establish the models of type 2 DM and cerebral ischemia, and examined the changes in body weight and blood glucose levels. Based on the structural changes in the retina and the retinal expression of VEGF, the protective mechanisms of ischemic PC on the DR of tree shrews were revealed to be associated with reduced retinal VEGF expression. Our study provides additional experimental basis for using ischemic PC in clinical prevention and treatment of DR.

## 2. Materials and Methods

### 2.1. Animals and Grouping

Healthy adult male tree shrews (body weight 130 ± 30 g) were provided by the National Primate Research Center of the Institute of Medical Biology, Chinese Academy of Medical Sciences (contract no. SYXK-Dian-K2013-0001). The tree shrews were maintained under standard feeding conditions (one animal in one cage, free access to chow and water) in an animal room with artificial light cycles (light time 08: 00-20: 00), room temperature (25 ± 3)°C, and humidity 40%-60% in the animal center of Kunming Medical University, Kunming, Yunnan Province, China. The chow for tree shrews was from Chengdu Dashuo Experimental Animal Co., Ltd., China. The formula of high-fat diet was as follows: basic chow supplemented with 1% cholesterol, 0.1% sodium cholate, 10% lard, 5% egg yolk powder, and whole milk powder.

**Figure 1:**
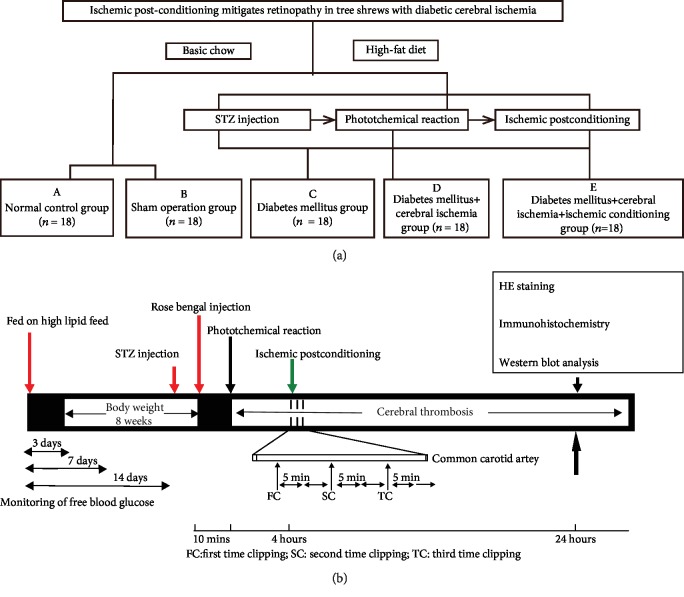
The outline of animal grouping and experimental designs. (a) According to the model to be established and the interventions to be taken, tree shrews were randomly assigned into 5 groups with initial 18 animals in each group, as follows: (a) normal control group (control); (b) sham operation group (sham); (c) diabetes mellitus group (DM); (d) diabetes mellitus+cerebral ischemia group (DM+IS); (e) diabetes mellitus+cerebral ischemia+ischemic conditioning group (DM+IS+PC). Tree shrews in the normal control and sham operation groups were fed with basic chow, and tree shrews in the other 3 groups were fed a high-fat diet for 8 weeks. (b) A schematic diagram shows the timeline and treatments. Detailed experimental procedures are described in the Materials and Methods section.

According to the model to be established and the interventions to be taken, tree shrews were randomly assigned into 5 groups with initial 18 animals in each group, as follows: A, normal control group; B, sham operation group; C, diabetes mellitus group; D, diabetes mellitus+cerebral ischemia group; and E, diabetes mellitus+cerebral ischemia+ischemic conditioning group (Figure 1(a)). Tree shrews in the normal control and sham operation groups were fed with basic chow, and tree shrews in the other 3 groups were fed a high-fat diet for 8 weeks. Sham operation referred to the operation of the tree shrews under the same conditions as other experimental groups, such as replacing the streptozotocin (STZ) and Rose Bengal sodium salt required for inducing the disease model with physiological saline and performing the same surgical operation on the corresponding part of the right brain but without photochemical induction. All procedures involving experimental animals were approved by the Laboratory Animal Ethics Committee of Kunming Medical University.

### 2.2. Preparation of Solutions for Modeling

The citric acid and sodium citrate solution was made by mixing citric acid, sodium citrate solution (Tianjin Guangfu Technology Development Co., Ltd., China), and distilled water at a ratio of 1 : 1 : 32 to achieve a concentration of 0.01 mol/L. The pH value was adjusted to 4.2, and each time, only the freshly generated solution was used for dissolving streptozotocin. Streptozotocin (Sigma-Aldrich, USA) was made as a 2% solution in the ice-cold citric acid and sodium citrate solution and stored in the dark. Rose Bengal sodium salt (4,5,6,7-tetrachloro-2,4,5,7-tetraiodofluorescein sodium salt; Sigma-Aldrich, USA) was formulated to a concentration of 1.5% solution, and stored with protection from light exposure at 4°C for later use.

### 2.3. Type 2 Diabetes Model in Tree Shrews

After feeding a high-fat diet for 8 weeks, tree shrews were weighed on the day of modeling and deprival of food and water for 8 hours (Figure 1(b)). Anesthesia was performed by injecting intraperitoneally 1% thiopental at a dose of 200 mg/kg body weight. Diabetic modeling was established by intravenous injection of 2% streptozotocin (100 mg/kg body weight). The normal control group was injected with citric acid-sodium citrate buffer (0.01 mol/L, pH 4.2) at a dose of 100 mg/kg body weight. The animals were observed daily for food intake, drinking water intake, and body weight changes. If the blood glucose concentrations were greater than 16.7 mmol/L at all the three time points (3, 7, and 14 days after modeling), the animals were considered diabetic. The tree shrews in the experimental group that did not meet this standard were supplemented with another STZ treatment at the same dose. If the animal still failed to meet the standard, it was not included in the follow-up study. The STZ-induced diabetic tree shrews after modeling were not treated with hypoglycemic therapy and their hyperglycemia state maintained for 4 weeks, in order to mimic the chronic hyperglycemia process and better simulate the pathogenesis of clinical chronic complications of diabetes. After the diabetes model was successfully established, the animals were stratified by blood glucose and randomly divided into three groups: diabetes mellitus group, diabetes mellitus+cerebral ischemia group, and diabetes mellitus+cerebral ischemia+ischemic conditioning group (Figure 1(a)).

### 2.4. Photochemically Induced Cortical Thrombotic Cerebral Ischemia Model in Tree Shrews

The diabetic tree shrews were fed a high-fat diet for 8 weeks and used for the thrombotic local cerebral ischemia model. The tree shrews were weighed and anesthetized through intraperitoneal injection of 1% thiopental solution at a dose of 200 mg/kg body weight. After being fixed in a prone position on the operating table, the animals were disinfected on the right top skin. An incision about 1 cm long was made between the right ear and the right eye, and the diaphragm was separated to expose the skull. At this time, the intracranial blood vessels were visible through the skull. A sterile aluminum piece was embedded in the incision. The center of the aluminum piece had a circular hole with a diameter of 0.5 cm. After the area around the aluminum sheet was covered, the Rose Bengal sodium salt was injected into the femoral vein. After 10 minutes of circulation, the SQ-III cerebral thrombosis device (patent number: ZL201420068737.2) was used to permeate light at the skull surface through the above circular window. The special light beam with a light intensity of 1.0 W/cm^2^ and a central wavelength (*λ*) of 560 nm can photochemically react with the Rose Bengal sodium salt and oxygen in the cerebral blood vessels to induce endothelium damage and thrombosis. During the experiment, the surface temperature of the skull was maintained at 36 ± 1°C, and the time was electrically controlled (15 min). After the operation, the aluminum piece was taken out, and the incision was disinfected and sutured layer by layer. Temperature was maintained until the animal was awakened, and they were placed in the cages for further observation.

### 2.5. Ischemic Postconditioning in Tree Shrews

The diabetic tree shrews were anesthetized again at 4 h after the photochemically induced thrombosis modeling (Figure 1(b)). After being fixed at a supine position, the animal was subjected to a longitudinal incision in the middle of the neck, and the right common carotid artery was isolated. The artery was clamped with a noninvasive clip on the upper edge of the thyroid cartilage to block the common carotid artery for 5 min. Then the arterial clip was removed to allow blood flow for 5 min. These 3 cycles of clamping and release were alternately performed to recapitulate the processes of ischemic conditioning. The animal was sutured for neck wound and observed for the clinical characteristics of cerebral ischemia in the initial 24 hours. The tree shrews in the sham operation group were only subjected to the isolation of right common carotid artery but without clamping of artery.

### 2.6. Histology by Hematoxylin and Eosin (H&E) Staining

After anesthetization with intraperitoneal injection of 1% thiopental into the animal, the right eyeball was quickly removed. The eyeball with a central puncture in the cornea was immersed in 10% neutral formalin for fixation. After 12 hours, it was washed with distilled water, and the eyeball was cut along the limbus to remove the eye. After removing vitreous body, the eyecup containing retina was soaked in 10% neutral formalin and fixed again for 24 h. The slices were dewaxed, hydrated, and stained with hematoxylin, 1% hydrochloric acid ethanol, and 0.5% eosin dye for 1-3 min, respectively. After washing with distilled water, gradient alcohol dehydration, and clearance and mounting, the slices were observed under a light microscope for morphological changes of the retina.

### 2.7. Immunohistochemistry Staining

The prepared sections were subjected to antigen retrieval and blocking following the standard protocol. The sections were incubated with primary Abs against VEGF (Cat. no. BIO11554; Beacom, Inc., Birmingham, England), at 4°C overnight. After washing with cold PBS, the sections were incubated with HRP-conjugated goat anti-rabbit secondary antibody (9001 SP link Detection Kits, Biotin-Streptavidin HRP Detection Systems) at room temperature for additional 30 min. The sections incubated with normal rabbit IgG were considered negative controls. An inverted fluorescence microscope (Nikon, Eclipse Ti-S, Japan) was used for visualization (magnification: 400x), and brown staining was considered a positive signal. Five individual fields per slide were selected for the evaluation of staining intensity.

### 2.8. Western Blot

After washing with cold PBS, retinal tissues were ground and lysed in radioimmunoprecipitation assay lysis buffer (RIPA; Sigma-Aldrich) containing a protease and phosphatase inhibitor cocktail on ice for 30 minutes. After centrifugation at 13000 g for 20 minutes at 4°C, proteins in the supernatants were quantified and equal amounts of total proteins were loaded. Samples were separated by 10% SDS-PAGE, transferred to polyvinylidene difluoride (PVDF) membranes, and incubated with the primary anti-VEGF D antibody (Cat. no. ab155288; Abcam, Cambridge, MA, USA) overnight. The antibody for the loading control *β*-actin (Cat. No. HC201) was from TransGen Biotech, Inc. (Beijing, China). The secondary horseradish peroxidase- (HRP-) conjugated anti-rabbit IgG (Cat. No HS101, TransGen Biotech, Inc.) antibody was used. Proteins of interest were visualized using enhanced chemiluminescence kit (EMD Millipore, Burlington, MA, USA). The band intensities were quantified by densitometry using ImageJ software (version 1.49; National Institutes of Health, Bethesda, MD, USA).

### 2.9. Statistics

Statistical analysis was performed using SPSS 17.0 software (IBM, USA). Quantitative data were described by the mean ± standard deviation, and differences between groups were compared using one-way analysis of variance (ANOVA). A *P* value < 0.05 was considered statistically significant.

## 3. Results

### 3.1. Effects of Diabetes Induction, Thrombotic Cerebral Ischemia, and Ischemic Conditioning on Body Weight and Blood Glucose Levels in Tree Shrews

In our animal models, tree shrews were fed a high-fat diet and treated with STZ (Figure 1). The purpose of high-fat diet feeding is to induce insulin resistance and to photocopy more risk factors such as high-fat and high-cholesterol levels which render the model with closer clinical characteristics of metabolic diseases and cerebrovascular diseases. The ratio of successful modeling in tree shrews through inducing diabetes with STZ was 77% (27/35). The model group exhibited the common symptoms of diabetes, such as polydipsia, polyphagia, and polyuria. The sham operation group and the normal control group had normal intake of drinking water and urine output. These animals demonstrated stable mood, quick response, and brown and shiny body hair. The STZ-induced diabetic tree shrews in the other 3 groups had significantly increased volumes of drinking water and urine, and their body hair color was yellow and dull.

**Figure 2:**
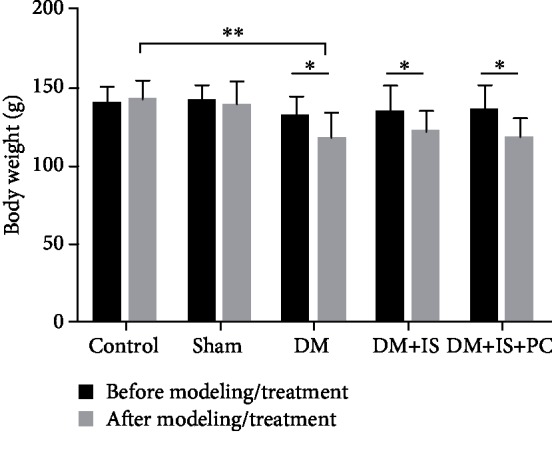
Comparisons of body weight in all groups of tree shrews before and after modeling/treatment. As shown in Figure 1, tree shrews were modeled for type 2 diabetes with STZ injection, and their hyperglycemia state maintained for another 4 weeks. Then, some of the DM tree shrews were subjected to photochemically induced cortical thrombotic cerebral ischemia and subsequent ischemic postconditioning. At 24 hours after ischemic PC, the relevant parameters were evaluated. *n* = 9 for each group; ^∗^*P* < 0.05 and ^∗∗^*P* < 0.01, between the indicated groups.

There were no differences in body weight and blood glucose levels among the groups before modeling. However, the animals with STZ-induced diabetes displayed significant changes in these parameters. After modeling, there was no difference in body weight between the diabetes group, the diabetes+cerebral ischemia group, and the diabetes+cerebral ischemia+the ischemic postconditioning group, but the body weight in these groups was significantly lower than that of the sham operation group and the normal control group (Table 1 and Figure 2). The difference of body weight between the diabetic tree shrews and the animals in the control or sham operation groups was statistically significant (*P* < 0.05). There was no difference in animal body weight between the sham operation group and the normal control group, either before or after modeling (Table 1 and Figure 2).

**Figure 3:**
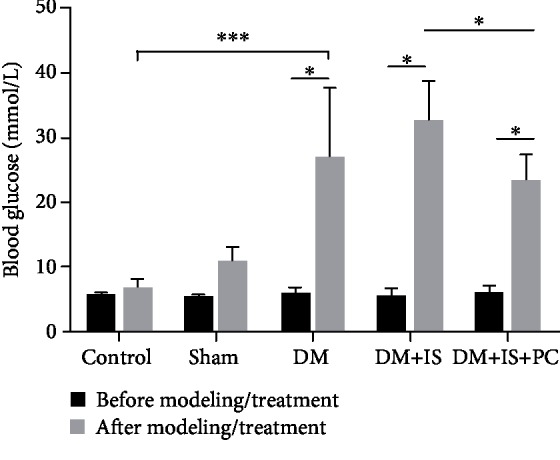
Comparisons of blood glucose levels in all groups of tree shrews before and after modeling/treatment. The experimental procedures were the same as stated in Figures 1 and 2, except that the levels of blood glucose were compared. *n* = 9 for each group; ^∗^*P* < 0.05 and ^∗∗∗^*P* < 0.001, between the indicated groups.

There was no difference in blood glucose levels between the sham operation group and the normal control group (Table 2 and Figure 3), suggesting that the surgical operations had no significant effects on the elevation of blood glucose levels. The animals in the diabetes group, the diabetes+cerebral ischemia group, and the diabetes+cerebral ischemia+postconditioning group had significantly higher blood glucose levels than the animals in the sham operation group and normal control group, which implied that the establishment of diabetes relied mainly on the destruction of the pancreas by STZ (Table 2 and Figure 3). Notably, the animals in the diabetes+cerebral ischemia+ischemic postconditioning group displayed much lower blood glucose levels that the animals in the diabetes+cerebral ischemia group (Table 2 and Figure 3). These results indicated that the postischemic adaptation treatment had protective effects on diabetic tree shrews, which was beneficial to alleviate the large fluctuations of blood sugar in the body.

### 3.2. Effects of Diabetes Induction, Thrombotic Cerebral Ischemia, and Ischemic Conditioning on Morphological Changes of the Retina in Tree Shrews

**Figure 4:**
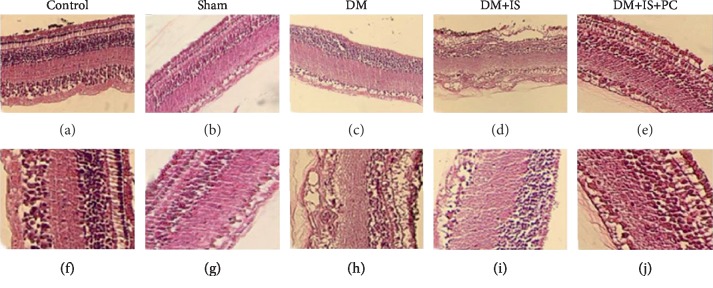
Histopathological features of retinal tissue in all groups of tree shrews. The experimental procedures were the same as stated in Figures 1 and 2. The retinal tissues of tree shrews in all the groups were harvested to examine the pathological structure changes of retina by H&E staining. (a–e) ×200 magnification; (f–j) ×400 magnification.

To further confirm the beneficial effects of ischemic postconditioning, we examined and compared the pathological changes of the retina in tree shrews that received various treatments by H&E staining. Similar as the structure of human retinal tissue, the retina of tree shrews in the control normal group (Figure 4(a) and 4(f)) and the sham operation group (Figure 4(b) and 4(g)) had clear inner limiting membrane, sparse nerve fiber layers. The ganglion cells were arranged in a single layer, and their nuclei were large, round or elliptical with lighter staining and neat arrangement. The inner plexiform layer was thicker and looser, showing a distinct network structure. The inner nuclear layer was composed of 4-5 layers of cells with slightly larger nucleus and slightly deeper staining. The outer plexiform layer was obviously thinner than the inner layer. The outer layer of retina was composed of 8-10 layers of cells with small nucleus and strong staining. These cells were in tight arrangement, and the outer membrane boundary was unclear. The cone and rod cell layers were well arranged, and the pigment epithelial cells were composed of monolayer cells (Figures 4(a), 4(b), 4(f), and 4(g)).

For the tree shrews in the diabetes group, the retina layers were thinned, and the number of cells in each layer was significantly reduced. Markedly, the arrangement of cells was disordered. The cells in the inner and outer nuclear layers were swollen, and the interstitial cells were obviously edematous (Figures 4(c) and 4(h)). In the diabetes+cerebral ischemia group, the retinal cells of tree shrews were disorderly arranged. The outer plexiform layer became thinner. The reticular structure became loose, and the inner granular layer cells were arranged in disorder. In the inner and outer granular layers, the ganglion cell layer and the nerve fiber layer, the capillary lumen was thickened with obvious telangiectasia. Red blood cells were visible in the lumen, and some red blood cells leaked into the extravascular tissues (Figures 4(d) and 4(i)). In the diabetes+cerebral ischemia+ischemic postconditioning group, the cells in the retina were well arranged. Although the cells were slightly edematous, the retinal thickness was close to that of the normal control group (Figures 4(e) and 4(j)). Taken together, these results suggested that ischemic conditioning significantly mitigated the pathological changes of diabetic retinopathy.

### 3.3. Effects of Diabetes Induction, Thrombotic Cerebral Ischemia, and Ischemic Conditioning on VEGF Expression in Retina of Tree Shrews

**Figure 5:**
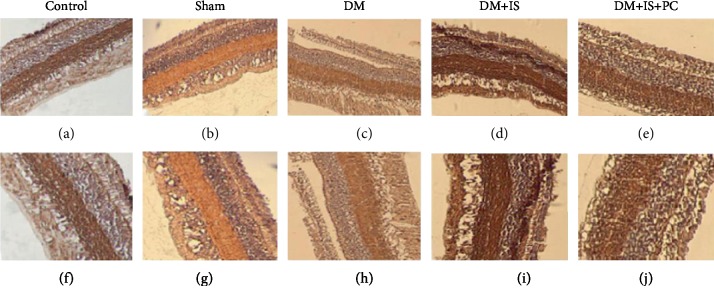
Immunohistochemical staining of VEGF in retinal tissues in all groups of tree shrews. The experimental procedures were the same as stated in Figures 1 and 2. The retinal tissues of tree shrews in all the groups were harvested to examine the expression levels of VEGF in the retina by immunohistochemical staining. (a–e) ×200 magnification; (f–j) ×400 magnification.

**Figure 6:**
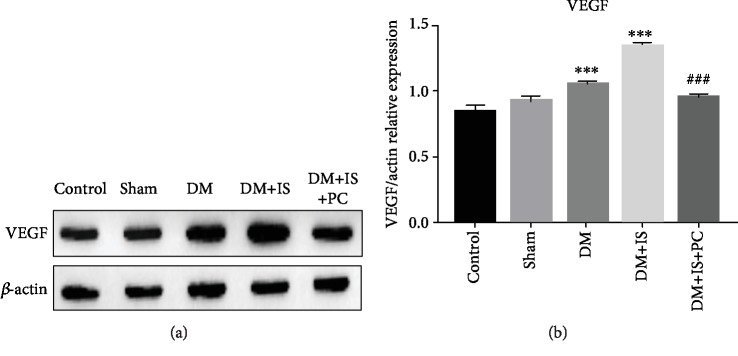
The retinal expression of VEGF in all groups of tree shrews was quantitated by Western blot assay. (a) The experimental procedures were the same as stated in Figures 1 and 2. Representative images show the blots of VEGF and the loading control *β*-actin from retinal tissues in the indicated groups. (b) The relative expression level of retinal VEGF was quantitated through comparing the densitometry-based band intensity of VEGF to that of *β*-actin. Data were summarized from three independent experiments with similar results. ^∗∗∗^*P* < 0.001, compared with the control group; ^###^*P* < 0.001, compared with the DM+IS group.

To further reveal the molecular mechanisms underlying the protective effects of ischemic postconditioning, we evaluated and compared the expression levels of VEGF in the retina of tree shrews that received various treatments by immunohistochemistry staining. The expression of VEGF in the retina of tree shrews from the normal control group and the sham operation group was predominantly distributed in the ganglion cell layer. The inner nuclear layer also displayed a small amount of VEGF expression, showing a weak positive staining (Figures 5(a), 5(b), 5(f), and 5(g)). The expression levels of VEGF in the retina from the diabetes group and the diabetes+cerebral ischemia group were evidently higher than those from the control or sham operation groups, while the diabetes+cerebral ischemia group had the most expression of retinal VEGF (Figures 5(c), 5(d), 5(h), and 5(i)). It is worth noting that VEGF expression in the retina of the tree shrews from the diabetes+cerebral ischemia+ischemic group significantly reduced compared with that from the diabetes+cerebral ischemia group (Figures 5(e) and 5(j)). Furthermore, the altered expressions of VEGF among these groups were validated by Western blot assays using tree shrew retinal tissues (Figure 6(a)), and the similar trends on retinal VEGF expression levels were observed (Figure 6(b)).

## 4. Discussion

Diabetic retinopathy is a common and serious complication of diabetic microangiopathy. As a chronic process that develops over a long period of time, it leads to irreversible visual impairment or complete blindness [5, 14]. This study determined the effects of ischemic postconditioning on diabetic retinopathy and the retinal expression of VEGF from tree shrews with diabetic cerebral ischemia and provided experimental and theoretical evidence for future nonpharmacological treatment of clinical diabetes patients with cerebral ischemic retinopathy.

We observed significantly elevated expression of retinal VEGF in tree shrews with induction of diabetes by STZ injection, while the tree shrews with additional cerebral ischemia demonstrated more retinal VEGF expression. This further validated the roles of ischemia in promoting DR progression, whereas ischemic postconditioning downregulated retinal VEGF expression in tree shrews that received both STZ administration and cerebral ischemia challenge, indicating the protective roles of PC. Contrary to our experiment results in tree shrews, some studies demonstrated that ischemia or hypoxia conditioning was able to increase VEGF expression [25, 26]. Probably, this discrepancy was due to the disease models with different species of animals and different experimental strategies for ischemia or hypoxia conditioning. We chose tree shrews largely because they closely resemble human ocular anatomy and pathologic features of the human retinal diseases [27]. For example, the retina from a tree shrew is dominated by cone cells and tree shrews have good color vision and high color discrimination accuracy and are sensitive to light and darkness [28–30]. Moreover, the phylogenetic tree analyses demonstrate that tree shrew is in closer proximity to primates than rodents [31, 32]. Therefore, the tree shrew model in this study bridges a gap between the established rodent and primate models of diabetic retinopathy. Since tree shrew is a species closer to humans than rodents like rats and mice, it is plausible that our study might be more faithfully transferred to clinics.

Compared with the DM group, the DM+cerebral ischemia group had significantly increased expression of VEGF in the retinal tissue of tree shrews (*P* < 0.01), suggesting that cerebral ischemia can enhance the expression of VEGF in diabetic tree shrew, while in the DM+cerebral ischemia+ischemic PC group, the thickness of the retina layers was close to that of normal retina and the morphology of pigmented cells was normal. Notably, the expression of VEGF was also significantly decreased (*P* < 0.01), suggesting that ischemic PC can prevent retinal ischemia and hypoxia and delay the development of DR. Consistent with our observation, the increase of retinal VEGF expression was also noticed in rats with DM, and ischemic conditioning or pulses could significantly decrease VEGF expression [19, 24].

The mechanisms of beneficial roles of PC may be related to the improvement of regional cerebral blood flow (rCBF) and inhibition of positive feedback regulation of VEGF expression by tissue hypoxia [33, 34]. The cells in the retina of DM patients can secrete and express VEGF, and it is also a specific region that is prone to be ischemic. Thus, it is speculated that the scope of DM retinal ischemia and hypoxia mainly focuses on the ganglion cell layer, inner plexiform layer, and inner nuclear layer. With the aggravation of the disease, once the DM patients have complications such as cardiovascular and cerebrovascular diseases, the scope of retinal hypoxia-ischemia can be gradually expanded and eventually results in the ischemia and hypoxia in the whole layer of the retina [33, 34]. Thus, ameliorating the syndrome of cardiovascular and cerebrovascular diseases in DM patients by ischemic conditioning or other approaches can also effectively mitigate the progression of DR.

Current investigations suggest that although PCs require more stringent execution times, postconditioning and preconditioning show equal benefits on cardioprotective and neuroprotective functions [35]. Since the operation of PC features with the easy-to-grasp timing and strong controllability and displays similar effects as the preconditioning in reducing the degree of reperfusion injury, there seems to be more clinical application values for ischemic PC in the therapy of diabetic retinopathy. In recent years, cerebral ischemic PC has gradually gained attentions, although its mechanisms are not fully understood. Multiple mechanisms, such as improvement on microcirculation [36, 37], inhibition of retinal cell apoptosis [38], and regulation of adenosine production [39], have been identified to be involved in the protective roles of PC. Diabetic retinopathy is a microvascular disease. Microcirculation is the basic unit of tissue blood perfusion, and it is the most susceptible to dysfunction. Retinal ischemia and hypoxia are closely related to microcirculatory disorders; therefore, improving microcirculation is an important means to maintain tissue blood perfusion. However, although ischemic PC provides a new option for the treatment of ischemic retinal disease, its mechanism needs further exploration. Although more work like optimization on the treatment time points and duration is needed to improve the therapeutic effects, our animal model established with tree shrews here provides a feasible and effective platform for further investigating the molecular mechanisms of ischemic PC in mitigating DR.

## 5. Conclusion

In summary, we established a tree shrew diabetic model through high-fat diet feeding combined with STZ injection in animals. The retinal expression of VEGF in tree shrews significantly increased with the aggravation of diabetes and cerebral ischemia, and ischemic postconditioning can effectively reduce the expression of VEGF in the retina of tree shrews with diabetic cerebral ischemia. Our preliminary exploring on establishing the tree shrew diabetic model and the protective roles of ischemic postconditioning supplements further experimental evidence for applying ischemic PC for DR therapy in clinics.

## Figures and Tables

**Table 1:** Comparisons of body weight in all groups of tree shrews.

	Control	Sham	DM	DM+IS	DM+IS+PC	*F*	*P*
	*n* = 9	*n* = 9	*n* = 9	*n* = 9	*n* = 9		
BW1	138.97 ± 9.61	140.49 ± 9.94	131.44 ± 11.82	134.32 ± 15.73	135.43 ± 14.74	0.748	0.565
BW2	141.15 ± 11.76	137.99 ± 14.36	116.53 ± 16.30	120.29 ± 13.82	117.12 ± 12.68	6.697	*P* = 0.001^∗∗^

Note: ^∗^*P* < 0.05; ^∗∗^*P* < 0.01; BW: body weight.

**Table 2:** Comparisons of fasting blood glucose levels in all groups of tree shrews.

	Control	Sham	DM	DM+IS	DM+IS+PC	*F*	*P*
	*n* = 9	*n* = 9	*n* = 9	*n* = 9	*n* = 9		
BG1	5.38 ± 0.52	5.17 ± 0.41	5.72 ± 0.96	5.35 ± 1.09	5.78 ± 1.09	0.820	0.52
BG2	6.52 ± 1.32	10.66 ± 2.24	26.75 ± 10.60	32.29 ± 6.08	23.04 ± 4.13	30.835	*P* = 0.001^∗∗^

Note: ^∗^*P* < 0.05; ^∗∗^*P* < 0.01; BG: blood glucose level.

## Data Availability

The data used to support the findings of this study are available from the corresponding author upon request.
